# Institutional Needs

**Published:** 1914-09-05

**Authors:** 


### Institutional Needs.

### MILK STERILIZER.

The milk problem has received more attention and excited more heart-searchings of recent months than ever, and so long as sterilization is considered the only available safeguard against impurity, the process by which this is carried out becomes of great importance. An ingenious method is embodied in the sterilizers manufactured by Aymard's Patent Milk Sterilizer Company, St. Matthew's Works, Ipswich. Its aim being to secure the effect of boiling without altering the taste of the milk. This is accomplished by placing the milk in a container surrounded by a jacket of steam generated by a boiler underneath. Burning the milk is therefore impossible, and the heat is satisfactorily transmitted. It is apparent, then, that the apparatus is simple, and therefore easy to clean, as well as efficient. It may be added that the steam required may be taken from the main kitchen supply, or gas burners can be fitted at no extra cost, while in cases where even gas may not be available a furnace for burning coal, coke, or wood can be fitted at the same price. The sterilizer is made in different sizes, and is used extensively in institutions which have found the sterilization to be satisfactorily carried out.

**Owing to the pressure upon our columns this week, we have been compelled to omit "The Institutional Worker" Supplement. This feature, however, will be resumed with our next issue, September 12.**

